# What do I do? Developing a taxonomy of chaplaincy activities and interventions for spiritual care in intensive care unit palliative care

**DOI:** 10.1186/s12904-015-0008-0

**Published:** 2015-04-15

**Authors:** Kevin Massey, Marilyn JD Barnes, Dana Villines, Julie D Goldstein, Anna Lee Hisey Pierson, Cheryl Scherer, Betty Vander Laan, Wm Thomas Summerfelt

**Affiliations:** Advocate Health Care, 3075 Highland Parkway, Downers Grove, IL 60515 USA

**Keywords:** Taxonomy, Spiritual care, Chaplaincy, Palliative care, Standard terminology

## Abstract

**Background:**

Chaplains are increasingly seen as key members of interdisciplinary palliative care teams, yet the specific interventions and hoped for outcomes of their work are poorly understood. This project served to develop a standard terminology inventory for the chaplaincy field, to be called the chaplaincy taxonomy.

**Methods:**

The research team used a mixed methods approach to generate, evaluate and validate items for the taxonomy. We conducted a literature review, retrospective chart review, focus groups, self-observation, experience sampling, concept mapping, and reliability testing. Chaplaincy activities focused primarily on palliative care in an intensive care unit setting in order to capture a broad cross section of chaplaincy activities.

**Results:**

Literature and chart review resulted in 438 taxonomy items for testing. Chaplain focus groups generated an additional 100 items and removed 421 items as duplications. Self-Observation, Experience Sampling and Concept Mapping provided validity that the taxonomy items were actual activities that chaplains perform in their spiritual care. Inter-rater reliability for chaplains to identify taxonomy items from vignettes was 0.903.

**Conclusions:**

The 100 item chaplaincy taxonomy provides a strong foundation for a normative inventory of chaplaincy activities and outcomes. A deliberative process is proposed to further expand and refine the taxonomy to create a standard terminological inventory for the field of chaplaincy. A standard terminology could improve the ways inter-disciplinary palliative care teams communicate about chaplaincy activities and outcomes.

## Background

Chaplains are increasingly seen as key members of interdisciplinary palliative care teams, yet what chaplains specifically do in terms of assessments, hoped for outcomes, and interventions remains poorly understood [1]. Chaplains lack a consistent way to describe their activities. Attempts have been made to develop inventories of chaplain activities and propose standard terminologies, yet none of these attempts were empirically based and none of these attempts has emerged as normative [2-6]. Chaplains perform a variety of interventions with therapeutic intent yet lack a unified and consistent naming set for these interventions which would better portray to the inter-disciplinary medical team what goals and results they strive to achieve.

Our study undertook to meet this identified [7] gap in the field of chaplaincy by building an inventory of chaplain activities through a series of mixed methods in which chaplains provided and refined their own terms and verbal preferences for their practice. This was executed in both patient care contexts and through qualitative steps involving groups of chaplains.

## Methods

A qualitative and quantitative approach was used to execute three phases of the study: item generation, validity, and reliability. The Advocate Health Care Institutional Review Board of our organization approved this study.

### Item generation

#### Literature and retrospective chart review

Four published inventories [2-5] of chaplain activities were reviewed by team members. The review criteria for inclusion included being published and employing research methodology. These inventories were judged by the team members to be the best previous efforts preceding this project. The team incorporated these inventories into a collective initial inventory. As the initial items were emerging from the literature review and the retrospective chart review, the chaplain researchers perceived three categories of “granularity". Some items were very specific concrete actions. Some items were more like goals or outcomes. Some items seemed like something in between concrete items and goals. As the study progressed and as is seen in the results, we began grouping the items into these three categories, which we named “interventions” for concrete items, “intended effects” for goals and outcomes, and “methods". These categories were later validated by the concept mapping phase described below. In the retrospective chart review phase, chaplain care data was taken from patient records (n = 1126 patient encounters) that had at least one interaction with a hospital chaplain and were also seen in the Intensive Care Unit (ICU). Patients who had the following Diagnosis Related Groups (DRG) were included: Intracranial Hemorrhage/Cerebral Infarction (DRG 65), Intracranial Hemorrhage Malignancy of Hepatobiliary System or Pancreas with Morbidity (DRG 435), Respiratory System Diagnosis with Ventilator Support (DRG 207–208), Septicemia or Severe Sepsis with or without Mechanical Ventilation 96+ Hours (DRG 870–711), and Simple Pneumonia and Pleurisy with Complication or Comorbidity (DRG 193). These DRGs were used in this step at the suggestion of the palliative care physician on our team to encompass patients mirroring the palliative care and ICU context that would follow in later steps.

### External validity

#### Focus group/key informant interviews

Board Certified Chaplains (BCCs) and Board Certified - Eligible Chaplains (BCC-Es), who contributed to the care of patients, (n = 27) participated in one of five focus groups conducted at five hospitals within our system. The chaplains were asked to complete four tasks based on their experiences within patient care to determine: which items could be categorized together, which items did not apply to their activities, which items were redundant and which new items should be included. Additionally, eight key informant interviews [8] approximating the focus group experience were conducted with chaplains in administrative positions.

### Construct validity

Self-observation and experience sampling methodology [9-11] was used to determine that we were creating a taxonomy that accurately reflected chaplain activities. Three chaplains at different sites made daily observations of their activities with palliative care patients, their family member(s) and the ICU care team using the activity list that was generated by the previous item generation phase. Electronic data collection was used by the three chaplains to record their activities.

#### Self-observation

Three Board Certified chaplains operating at three different though comparable acute care hospitals made daily observations of their activities with ICU palliative care patients, their family member(s), and the ICU care team using the activity list generated in the previous steps. Each chaplain self-selected activities to be recorded in an electronic collection tool (one to five per day). For each activity, they were asked to select the intervention performed, the intended effect and method corresponding to the intervention, and with whom the intervention was performed.

#### Experience sampling

Chaplains were paged at random intervals during each shift for 28 days to record their current activity, similar to the method pioneered by Larson & Csikszentmihalyi [9-11]. The chaplains were asked to record their activities within 15 minutes after receiving the page and were alerted by pager six times per shift to identify on the electronic tracking tool if they were engaged in spiritual care or another activity such as administrative duties or on personal time. If a spiritual care intervention was selected, they also recorded the intended effect from the inventory.

#### Concept mapping

Concept mapping is a group-oriented, decision-making process to develop a framework of stakeholders’ views for a specific topic [12]. BCCs were recruited from focus group participants and from other institutions participating in the grant opportunity that supported this project. Participants (n = 30) were asked to group the activities into three categories (intended effect, method, and intervention) after receiving training on the data collection tool. Participants were then given a list of activity items and asked to rate each item on a five point Likert-type scale for frequency of use and importance.

### Reliability

#### Inter-rater reliability sessions

Vignettes of chaplain activities portraying the care of patients, family member(s), and care team were created from real-life chaplain examples. All vignettes were voice-acted and audio recorded by members of the research team so participants in each session would respond to a standardized vignette presentation. Participants were provided with a list of the taxonomy items divided into intended effect, method, and intervention, as well as a transcript of the vignettes. For each vignette, participants listened to the audio recording of the vignette while reading the transcript, if desired, and identified a Spiritual Care Plan (SCP). A SCP is an assembly of an intended effect, method, and intervention encompassing spiritual care provided.

**Figure 1:**
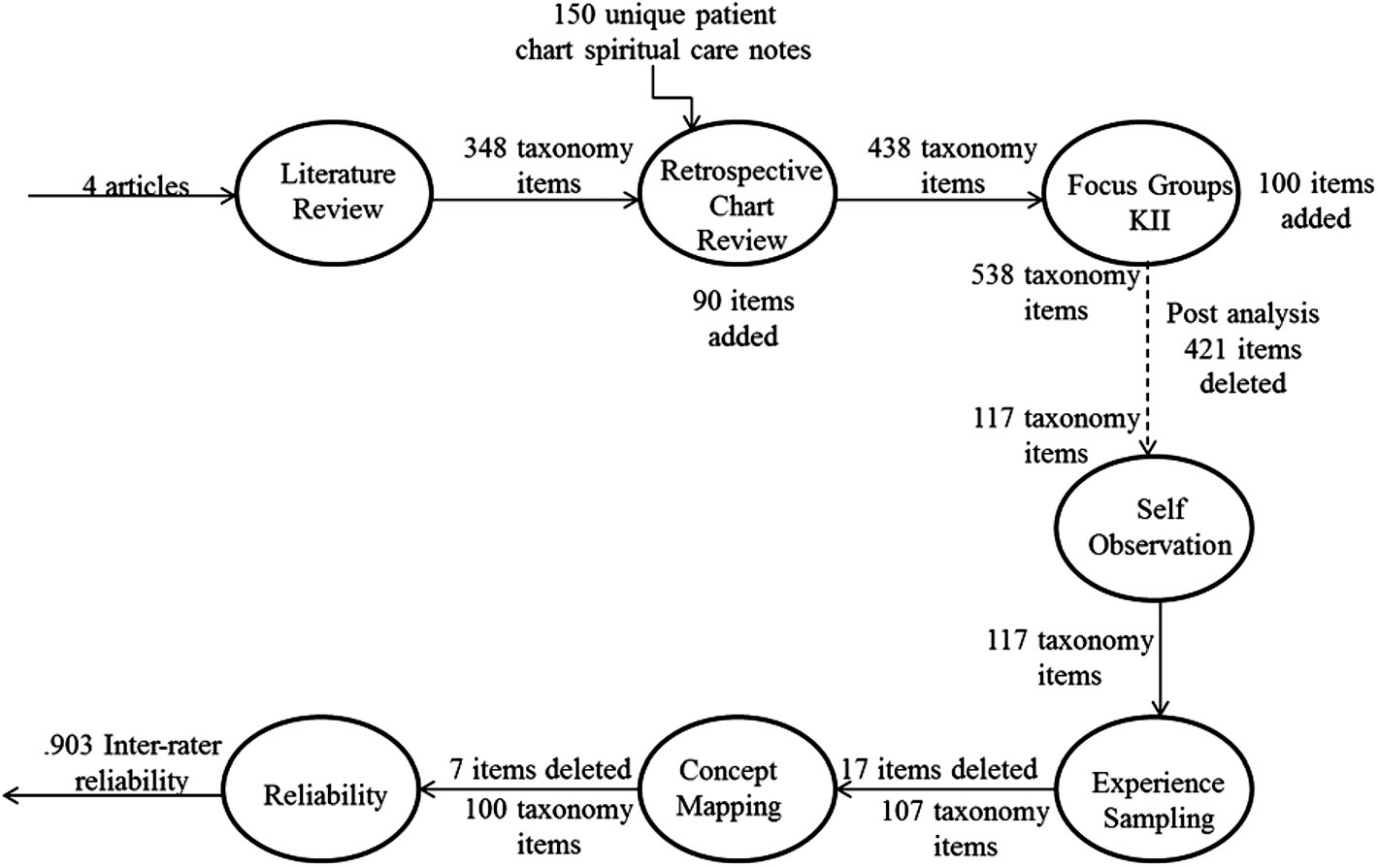
**Summary of methods used and taxonomy development.**

Figure 1 provides a high level view of the methods process.

### Data analyses and statistics

Counts and percentages were used for the patient chart review, chaplain focus group, self-observation and experience sampling data, and the concept map Likert-type ratings of frequency of use and importance are displayed as means with standard deviations. Inter-rater reliability was assessed using the intra-class correlation (ICC) [13]. The ICC is an advanced correlation that allows for a random-effects model and estimates agreement among raters. Analysis was performed using SPSS®18 with the exception of analysis for concept clusters. The concept mapping software organized and displayed data using multidimensional scaling and cluster analysis displayed as maps which show the relationship between clusters created by the participants.

## Results

The research engaged 67 religiously, ethnically, and geographically diverse chaplains; with 24% of the chaplains participating in three or more research methods.

### Item generation

#### Literature and retrospective chart review

The first version of the taxonomy was generated from the 348 items abstracted from the literature review and clinical experience, with 122 items identified in the previously published inventories and 226 additional items generated by the primary research team, reflecting on their own chaplain experiences. Apparent redundancies were purposely retained in the literature review and retrospective chart review stages to allow later stages to express chaplain preferences. Each additional step would result in a new version built upon the previous steps.

From the population of 1126 cases that met selection criteria for chart abstraction, a random sample of 50 cases from each of three hospitals (n = 150) was selected for review to abstract free text from the progress notes in the charts in search of unique chaplaincy activities for the inventory. Review of the charts resulted in 261 activities recorded. This step generated 90 additional items for placement within the taxonomy, bringing the total number of items to 438.

### External validity

#### Focus groups/key informant interviews

The focus groups and key informant interviews generated, deleted, added and revised numerous items of the taxonomy. Session four of the focus groups added the most items (n = 57). Session one identified the most items as duplicates (n = 278), and session two identified the most items as chaplain specific interventions (n = 245). Table 1 provides all session data. The key informant interviews generated similar items to the focus group sessions. After data analysis, the focus groups and key informant interviews generated an additional 100 items and deleted 421 items as duplicate or similar items leaving 117 chaplaincy taxonomy items.

#### Self-observation

Each chaplain self-selected activities (n = 646 chaplain activities) performed throughout their work shift. For the interventions, 79% of the items were used and 100% of the items were used for methods and intended effects.

The top five interventions accounting for 28% of the responses included “Active listening” (n = 46, 7.1%), “Prayer for healing” (n = 36, 5.6%) and “Silent prayer at bedside”, “Facilitate story-telling”, and “Facilitate communication between patient and/or family member and care team” each with n = 33, 5.1%. The top three methods accounting for 36% of the responses were “Demonstrate caring and concern” (n = 89, 13.8%), “Provide emotional support” (n = 79, 12.2%), and “Collaborate with care team member” (n = 64, 9.9%). The top four intended effects accounting for 58.1% of the responses and included “Establish a relationship of care and support” (n = 142, 22.0%), “Aligning care plan with patient's values” (n = 134, 20.7%), and “Exploring hope” and “Journeying with someone in the grief process” each with n = 50, 7.7%.

#### Experience sampling

Three chaplains provided 244 data points accounting for their chaplaincy and non-chaplaincy activities. Analysis of the data showed that chaplains spent 56% of their time on administrative, documentation, or personal time and 44% of their time providing spiritual care. Of this 44% of time spent on spiritual care, 42% of the time was spent with patients, 36% with a family member, and 43% spent with the care team. The top three intended effects chaplains used during this step were “Aligning care plan with patient’s values” (26%); “Establish a relationship of care and support” (21%); and “Journeying with someone in the grief process” (19%).

#### Concept mapping

There were a total of 30 BCC and BCC-E participants, from nine Health Care Systems. Other demographic information of the participants included: 23% Non-Christian, 67% Christian, 57% female, 73% Caucasian, and 80% of the chaplains identified hospitals as their work setting. The average years of chaplain experience across the participants was 15 years. The top ten frequency and importance rated items are provided in Table 2.

**Table 1:** **Focus group results**

**Session #**	**# of Items added to taxonomy**	**# of items identified as duplicates**	**# of items identified as Chaplain interventions**
1	13	278	173
2	10	219	245
3	7	235	210
4	57	125	198
5	13	271	180

**Table 2:** **Concept mapping results**

**Top 10 frequency rated items**	**Frequency rating**	**Top 10 importance rated items**	**Importance rating**
Active listening	5	Active listening	5
Provide a pastoral presence	5	Demonstrate caring and concern	4.9
Demonstrate caring and concern	4.9	Provide a pastoral presence	4.8
Preserve dignity and respect	4.9	Preserve dignity and respect	4.8
Remain open to patient’s beliefs	4.9	Collaborate with care team member	4.8
Collaborate with care team member	4.8	Build rapport and connectedness	4.8
Demonstrate acceptance	4.8	Establish a relationship of care and support	4.8
Build rapport and connectedness	4.8	Demonstrate acceptance	4.7
Provide emotional support	4.8	Provide emotional support	4.7
Establish a relationship of care and support	4.8	Provide support	4.7

### Content validity

Participant categorizations were compared to the categories established for the experience sampling data collection. Categorizations were compared between the data collection steps and each item was coded as congruent (same categorization in experience sampling and concept mapping) and or incongruent (different categorization in experience sampling and concept mapping). Of the 107 activities, 72 were congruent (67%) and 35 activities were incongruent (33%). A thorough review of all data collected and group discussion was used to resolve incongruent results and 19 (54%) of the 35 incongruent activities retained the concept mapping categorization with two activities (6%) removed from the activity list.

### Reliability

Twenty-seven BCCs, BCC-Es and chaplain residents (student chaplains engaged in a year-long training program) participated in five inter-rater reliability sessions at four hospitals within our system, plus one hospital within the region from a different health care system to increase sample diversity. Fifty different vignettes from actual chaplaincy encounters with an associated correct response were used during each session. Fifteen practice vignettes were used to familiarize participants with intended effects, methods and interventions used to describe why and how a chaplain performed an activity. Thirty five different vignettes were used for the inter-rater reliability assessment.

Upon completion of the data gathering and analysis, an inter-rater reliability of 0.903 was achieved based on an inter-rater reliability testing process. This was achieved without the elimination of any participant responses or testing questions. We note that there was one participant who scored 66%, four participants who scored 97% and two questions had less than a 50% correct response rate.

## Discussion

### Taxonomy description

Following the completion of all the generation, evaluation, and validation steps, the resulting taxonomy consisted of 100 items, presented in Table 3. The resulting inventory is practical, robust, and yet economical. While additional efforts are required to address remaining gaps and limitations, this taxonomy of chaplaincy activities provides a strong foundation for the sorely needed normative inventory of chaplaincy activities and a starting place for a standardized chaplaincy language.

**Table 3:** **The chaplaincy taxonomy**

**Intended effects**	**Methods**	**Interventions**	
Aligning care plan with patient’s values	Accompany someone in their spiritual/religious practice outside your faith tradition	Acknowledge current situation	Facilitate closure
Build relationship of care and support	Assist with finding purpose	Acknowledge response to difficult experience	Facilitate communication
Convey a calming presence	Assist with spiritual/religious practices	Active listening	Facilitate communication between patient and/or family member and care team
De-escalate emotionally charged situations	Collaborate with care team member	Ask guided questions	Facilitate communication between patient/family member(s)
Demonstrate caring and concern	Demonstrate acceptance	Ask guided questions about cultural and religious values	Facilitate decision making
Establish rapport and connectedness	Educate care team about cultural and religious values	Ask guided questions about faith	Facilitate grief recovery groups
Faith affirmation	Encourage end of life review	Ask guided questions about purpose	Facilitate life review
Helping someone feel comforted	Encourage self care	Ask guided questions about the nature and presence of God	Facilitate preparing for end of life
Journeying with someone in the grief process	Encourage self reflection	Ask questions to bring forth feelings	Facilitate spirituality groups
Lessen anxiety	Encourage sharing of feelings	Assist patient with documenting choices	Facilitate understanding of limitations
Lessen someone’s feelings of isolation	Encourage someone to recognize their strengths	Assist patient with documenting values	Identify supportive relationship(s)
Meaning-Making	Encourage story-telling	Assist someone with Advance Directives	Incorporate cultural and religious needs in plan of care
Mending broken relationships	Encouraging spiritual/religious practices	Assist with determining decision maker	Invite someone to reminisce
Preserve dignity and respect	Explore cultural values	Assist with identifying strengths	Perform a blessing
Promote a sense of peace	Explore ethical dilemmas	Bless religious item(s)	Perform a religious rite or ritual
	Explore faith and values	Blessing for care team member(s)	Pray
	Explore nature of God	Communicate patient’s needs/concerns to others	Prayer for healing
	Explore presence of God	Conduct a memorial service	Provide a religious item(s)
	Explore quality of life	Conduct a religious service	Provide access to a quiet place
	Explore spiritual/religious beliefs	Connect someone with their faith community/clergy	Provide compassionate touch
	Explore values conflict	Crisis intervention	Provide Grief Processing Session
	Exploring hope	Discuss concerns	Provide grief resources
	Offer emotional support	Discuss coping mechanism with someone	Provide hospitality
	Offer spiritual/religious support	Discuss frustrations with someone	Provide religious music
	Offer support	Discuss plan of care	Provide sacred reading(s)
	Setting boundaries	Discuss spirituality/religion with someone	Provide spiritual/religious resources
		Ethical consultation	Respond as chaplain to a defined crisis event
		Explain chaplain role	Share words of hope and inspiration
		Facilitate advance care planning	Share written prayer
			Silent prayer

### The taxonomy

As the taxonomy emerged, we tried out a variety of patterns of how to group and assemble items to represent spiritual care interactions. A pattern that emerged was one in which items can be assembled to represent a plan of progression including specific actions and the effects of those actions. The taxonomy items can be grouped and associated together in nearly infinite combinations to develop a grouping we have come to call a “pathway;” which is the assemblage of an Intended Effect – Method – Intervention. A pathway or pathways make up a Spiritual Care Plan which is developed in response to the identified spiritual care needs surfaced in a Spiritual Assessment. A variety of methods of Spiritual Assessment are current [14], which were out of the scope of our study. Spiritual Care Plans can be comprised of many different combinations ranging from a single intervention with numerous intended effects to numerous interventions with a single intended effect. Additionally, Intended Effect, Methods, and Interventions are provisional categories, and a case could be made for grouping the items into two categories such as Actions and Outcomes or Means and Ends.

Chaplains in our organization have found it more natural to assemble items and create pathways than to think about individual items in isolation. Chaplains in our organization have discussed assembling care plans of intended effects, methods, and interventions to match the spiritual care needs of particular patient populations. Some of these pathways common to palliative care of interest to readers of this journal include:

- Aligning care plan with patient’s values - Ask guided questions about cultural and religious values – Explore cultural values
- Preserve dignity and respect - Communicate patient’s needs/concerns to others – Collaborate with care team member
- Demonstrate caring and concern - Provide compassionate touch – Offer emotional support
- Faith affirmation - Perform a religious rite or ritual – Assist with spiritual/religious practices
- Meaning making - Invite someone to reminisce – Encourage self –reflection
- Establish Rapport and connectedness - Provide hospitality – Offer support
- Sense of Peace - Pray for healing – Exploring hope

### The taxonomy and palliative care

Previous studies in spirituality and palliative care have foreshadowed many of the themes that emerged in the intended effects list of the chaplaincy taxonomy. The item “Preserve dignity and respect” mirrors the important work on Dignity Therapy by Chochinov. [15] The item “Align care plan with patient’s values” mirrors a recent study showing connection between chaplains and hospice and palliative care use [16]. The emphasis on relationship; such as “Lessen someone’s feelings of isolation,” “Build rapport and connectedness,” and “Mending broken relationships” is important [17]. The multidimensional elements uplifted in the Special Report - Improving the Quality of Spiritual Care as a Dimension of Palliative Care: The Report of the Consensus Conference [18], which was a contributing inventory in the literature review for the chaplaincy taxonomy, are also well represented by the items that emerged. The taxonomy then is a recognizable inventory of chaplaincy activities known among palliative care teams.

The taxonomy could be used in a number of ways to enhance interdisciplinary team understanding of chaplaincy work. The taxonomy could form the basis of a clinical documentation set to standardize the descriptions chaplains share about their Spiritual Care Plan.

### Further resources and structuring of the taxonomy

We have completed a User’s Guide to the Taxonomy [19] that will assist chaplains and other palliative care members in using the taxonomy more reliably and consistently. The User’s Guide includes definitions of the specific taxonomy intended effects, methods, and interventions. The User’s Guide includes both the alphabetical listing of the taxonomy items found in Table 3 of this piece and another listing of the taxonomy items grouped into categories of similarity to assist a user in selecting items. For example, categories such as “Grief,” “Relationships” and “Spiritual/Religious Practice” group together items that pertain to each other on these themes.

### Limitations

While the taxonomy project involved chaplains from outside our health care system and outside the Chicagoland area, the majority of chaplains were from our system and area. Some questions of generalizability are raised by this fact. Further generalizability questions arise in that the taxonomy was developed mostly in a specific clinical context, namely ICU and Palliative Care. Inevitably there are gaps concerning interventions, methods, and intended effects perhaps unique to other contexts. For example, there may be items particular to pediatric contexts that are missing from this initial taxonomy. These generalizability concerns may have been addressed by the two steps, Focus Groups and Concept Mapping which included chaplains from other practice settings. Continued practice and use of the taxonomy in diverse geographic and organizational contexts can address these issues and may identify additional improvements to the taxonomy.

Some apparent redundancies and contradictions have survived the taxonomy formation process. For example, one intervention, “Ask guided questions about the nature and presence of God,” might be paired with two different methods, “Explore presence of God” and “Explore nature of God.” Either these two methods should be merged, or if it were judged that the two concepts are very distinct, then “Ask guided questions about the nature and presence of God” should be split out to two different interventions. As the taxonomy is used, questions such as this should be reevaluated and, if need be, the taxonomy should be updated.

## Conclusion

The adoption of a normative language among chaplains working on palliative care teams would be a welcome development in helping interdisciplinary teams better describe chaplaincy actions and goals. This taxonomy provides a firm foundation upon which such a normative language could be built. Further practice of this inventory by chaplains working on palliative care teams would continue to identify gaps and eliminate redundancy and refine and improve the inventory.

We propose testing this taxonomy in practice with all members of the palliative care team in order to facilitate communication and assessing outcomes. We encourage chaplains and other members of the palliative care team to identify redundancies and gaps and to further enrich the taxonomy for future use.

## Abbreviations

BCC: Board Certified Chaplain
BCC-E: Board Certified Chaplain-Eligible
DRG: Diagnosis Related Group
ICC: Intra-Class Correlation
ICU: Intensive Care Unit
KII: Key Informant Interviews
